# Chiral phase-transfer catalysis in the asymmetric α-heterofunctionalization of prochiral nucleophiles

**DOI:** 10.3762/bjoc.13.170

**Published:** 2017-08-22

**Authors:** Johannes Schörgenhumer, Maximilian Tiffner, Mario Waser

**Affiliations:** 1 Institute of Organic Chemistry, Johannes Kepler University Linz, Altenbergerstrasse 69, 4040 Linz, Austria

**Keywords:** amination, chlorination, fluorination, hydroxylation, thioetherification

## Abstract

Chiral phase-transfer catalysis is one of the major catalytic principles in asymmetric catalysis. A broad variety of different catalysts and their use for challenging applications have been reported over the last decades. Besides asymmetric C–C bond forming reactions the use of chiral phase-transfer catalysts for enantioselective α-heterofunctionalization reactions of prochiral nucleophiles became one of the most important field of application of this catalytic principle. Based on several highly spectacular recent reports, we thus wish to discuss some of the most important achievements in this field within the context of this review.

## Introduction

**Scheme 1:**
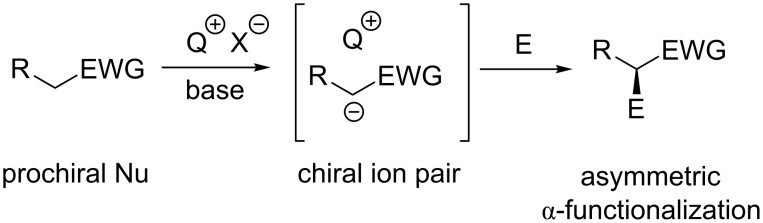
Generally accepted ion-pairing mechanism between the chiral cation Q^+^ of a PTC and an enolate and subsequent asymmetric α-functionalization with an electrophile (E).

In the 1960s and 1970s, the groups of Makosza, Starks, and Brändström pioneered the field of “phase transfer catalysis” by showing the beneficial effect of tetraalkyl-ammonium or -phosphonium salts to facilitate reactions between components that are present in two immiscible phases [1–6]. The introduction of this powerful concept had a lasting influence and significantly expanded the field of organic synthesis. The use of achiral onium salts as phase-transfer catalysts (PTCs) has now been well-established for many fundamental reactions under usually rather simple and easily scalable conditions [7–8]. Next to the systematic use of achiral onium PTCs (that has been investigated for half a century now), the development of asymmetric versions by using structurally well-defined chiral PTCs has attracted considerable interest too, on both, laboratory scale, and for industrial applications [9–24]. Different catalytically active structural motives have been used for this purpose so far, and amongst them chiral quaternary ammonium salts have become the most privileged class of chiral PTCs [9–20]. Following the pioneering reports of Wynberg et al. [25] and Merck scientists [26] who employed cinchona alkaloid-derived quaternary ammonium salts for asymmetric epoxidations [25] and the α-methylation of a phenylindanone derivative [26] in the late 1970s, early 1980s already, cinchona alkaloids remained the preferred chiral backbones for novel phase-transfer catalysts and applications thereof until the beginning of the 21st century. Pioneering contributions with these powerful catalysts were reported by the groups of O’Donnell [27], Lygo [28], and Corey [29]. The turn of the last millennium then witnessed an increasing interest in the design of new chiral ammonium salt phase-transfer catalysts, with remarkable contributions especially by Maruoka’s group, who developed very efficient and highly versatile chiral binaphthyl-based quaternary ammonium salt catalysts [12–14,30]. Alternative approaches by utilizing chiral backbones like, e.g., tartaric acid, biphenyls, or tricyclic ammonium salts were also heavily investigated [31–35], thus leading to an enormous recent progress in the field with respect to catalyst design and the development of new asymmetric applications. Besides quaternary ammonium salts, also chiral phosphonium salts [21,36], chiral (bis)guanidinium systems [22,27,37], chiral crown ethers [38–39], bifunctional onium salts [17,40–46], or even sulfonium salts [47–48] have been systematically developed very recently. Thus, it can be said without exaggeration that chiral phase-transfer or ion-pairing catalysis has become one of the major catalytic principles, which allows for a broad variety of powerful asymmetric applications that are only very difficult to achieve with other catalytic principles. One of the major advantages of all these chiral cation-based phase-transfer catalysts (Q^+^ X^−^) is their unique potential to control the reactivity of a broad variety of different prochiral nucleophiles (i.e., enolates) via formation of chiral ion pairs, which can then undergo stereoselective α-functionalization reactions with different electrophiles (Scheme 1).

A lot of different examples for such asymmetric α-functionalization reactions of prochiral nucleophiles under asymmetric chiral cation-based phase-transfer catalysis have been reported so far [9–22]. Besides C–C bond forming reactions like, e.g., α-alkylations, Michael additions, Mannich reactions, or aldol reactions, to mention the most prominent examples only, also asymmetric α-heterofunctionalizations like hydroxylations, halogenations, or aminations have become increasingly important over the last years [49–54]. Based on this recent interest, and also the tremendous progress made hereby, it is the main intention of this review to provide an illustrative overview about some of the most significant developments in the field of asymmetric α-heterofunctionalization reactions under chiral phase-transfer catalysis.

It is worth to note that besides chiral cation-based phase-transfer catalysts (like the aforementioned onium catalysts, which are by far the most commonly employed chiral PTCs), also the use of chiral anion-based PTCs has recently attracted a lot of attention and lead to a variety of highly versatile methods for asymmetric catalysis [24,55–63]. In contrast to chiral cation-based PTCs, which mainly operate through coordination and control of the nucleophile, these anionic PTCs usually coordinate cationic (and often hardly soluble) electrophilic reagents and this complementary strategy has as well been rather impressively used for different asymmetric heterofunctionalization reactions. Accordingly, also some selected very recent examples for the use of chiral anion-based PTCs for asymmetric α-heterofunctionalization reactions of prochiral nucleophiles will be discussed herein.

## Review

### α-Halogenations

Asymmetric α-halogenation reactions of prochiral nucleophiles became a very important topic and a variety of complementary C–X bond forming strategies have been introduced over the course of the last years [49–54]. On the one hand the hereby accessed products can be of significant interest with respect to biological or medical applications, which is especially the case for α-fluoro targets [64–65]. On the other hand, such α-halo carbonyl compounds may serve as valuable intermediates for further manipulations like, for example, enantiospecific nucleophilic displacement reactions [66–67].

#### α-Fluorinations

The stereoselective electrophilic α-fluorination of carbonyl compounds became a thoroughly investigated field over the course of the last 15 years [68–72]. A variety of different catalytic approaches, either relying on the use of chiral metal complexes, or chiral organocatalysts have been reported, and the use of chiral PTCs became a powerful strategy herein too [44,56–57,73–79].

**Scheme 2:**
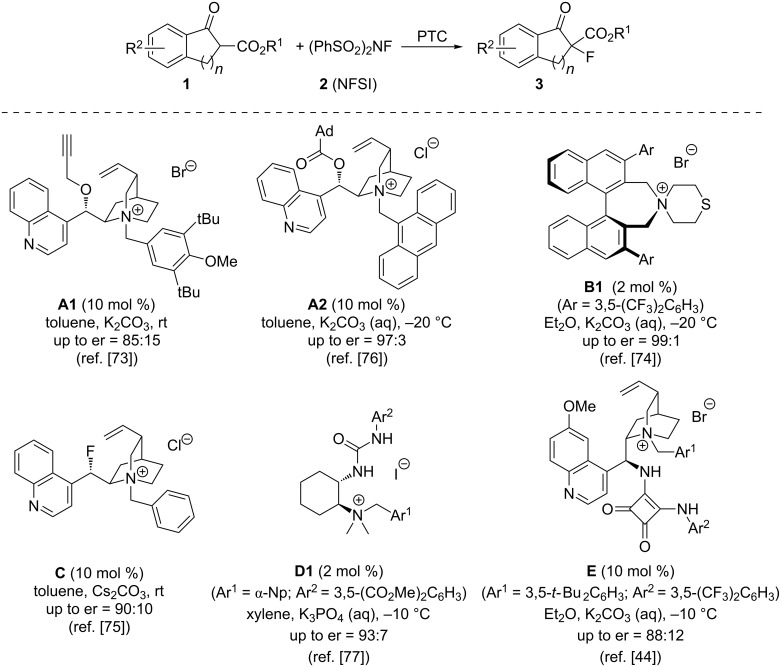
Reported asymmetric α-fluorination of β-ketoesters **1** using different chiral PTCs.

The seminal report on asymmetric phase-transfer-catalysed electrophilic α-fluorinations of prochiral carbonyl nucleophiles dates back to 2002, when Kim and Park described the first use of cinchona alkaloid-based quaternary ammonium salts **A** (i.e., derivative **A1**) for the enantioselective α-fluorination of β-ketoesters **1** by using *N*-fluorobenzenesulfonimide (NFSI, **2**) as the fluoride-transfer reagent [73] (Scheme 2). By applying biphasic conditions (using either K_2_CO_3_ or Cs_2_CO_3_ as solid bases) in the presence of 10 mol % **A1**, they were able to achieve enantiomeric ratios up to 85:15 under operationally simple room temperature conditions. After this pioneering report, it took around eight years until Maruoka’s group described the first highly enantioselective protocol for this reaction [74]. By using just 2 mol % of their trademark binaphthyl-based PTCs **B**, they were able to access a broad variety of differently functionalized α-fluoro-β-ketoesters **3** with excellent selectivities and in almost quantitative isolated yields. It should also be emphasized that this report still represents one of the most powerful and most selective chiral PTC-based approaches to access these important fluorinated targets nowadays.

A very interesting (mechanistic) investigation was reported by Gilmour’s group in 2012, who showed that substitution of the C9–OH group of the cinchona skeleton by fluorine has a pronounced effect on the conformation of these catalysts and that ammonium salt **C** can then be successfully used for the aforementioned asymmetric α-fluorination of ketoesters **1** [75]. A significant improvement for the use of classical cinchona-based PTCs **A** for such α-fluorination reactions was reported in 2013 by the groups of Meng and Lu [76]. By screening a variety of differently modified ammonium salts **A**, they found that the introduction of sterically demanding 1-adamantoyl esters in the 9-position of the cinchona alkaloids in combination with an anthracenylmethyl quaternary ammonium group (resulting in catalyst **A2**) allows them to carry out the syntheses of products **3** with high enantioselectivities [76].

Our group has over the last years been interested in the design and development of bifunctional (thio)urea/ammonium salt catalysts of the general structure **D** [45–46,77]. Besides testing these catalysts for asymmetric C–C bond-forming reactions [45–46], we also screened them for their potential to control the asymmetric α-fluorination of β-ketoesters **1** [77]. It was found that ammonium salt derivative **D1** allows for the synthesis of differently substituted ketoesters **3** with good to high enantioselectivities by using just 2 mol % of the bifunctional catalyst. Very interestingly, Shi’s group very recently also reported PEG-bound derivatives of these catalysts which performed more or less equally selective for this fluorination reaction [78]. The groups of Duan and Lin have also very heavily been engaged in the systematic design of highly active bifunctional cinchona alkaloid-based ammonium salt catalysts that contain an additional H-bonding donor [43–44]. Very impressively, they succeeded in synthesizing and testing the first squaramide-containing chiral PTCs **E**, which also turned out to be very promising catalysts for the asymmetric α-fluorination of compounds **1** [44].

**Scheme 3:**
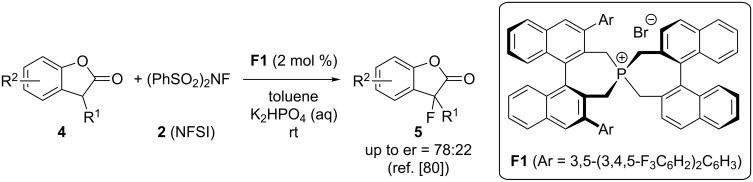
Asymmetric α-fluorination of benzofuranones **4** with phosphonium salt PTC **F1**.

The groups of Ma and Cahard have intensively investigated the use of chiral spirocyclic phosphonium salts **F** as phase-transfer catalysts for asymmetric α-heterofunctionalization reactions [79–80]. Hereby they also reported the fluorination of 3-substituted benzofuranones **4** by using NFSI (**2**) as the electrophilic F-transfer reagent [80]. The reaction could be carried out in excellent yields and with modest enantioselectivities for a rather broad substrate scope when using just 2 mol % of phosphonium salt **F1** under liquid/liquid biphasic conditions (Scheme 3).

**Scheme 4:**
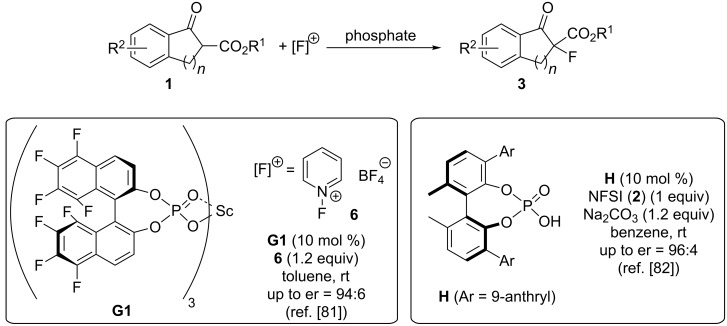
Asymmetric α-fluorination of **1** with chiral phosphate-based catalysts.

As already stated before, the use of chiral anionic PTCs became increasingly important over the last years and especially asymmetric fluorine-transfer reactions have been very extensively investigated by this strategy [56–57,81–83]. In 2006 already, Inanaga and co-workers reported the use of chiral scandium-containing organophosphates **G1** for the asymmetric α-fluorination of ketoesters **1** (Scheme 4) [81]. No clear mechanistic proposal was given in this interesting contribution and it turned out that scandium clearly outperforms other rare earth cations hereby, which makes it thus difficult to say if this is really a classical phase-transfer-catalysed reaction or rather a Lewis acid catalysed transformation controlled by a chiral counter anion (it is well known that chiral counter anions can control Pd-catalysed α-fluorinations [84]). Very interestingly, however, the authors clearly proved that the nature of the F-transfer reagent is crucial to obtain high selectivities. While NFSI or Selectfluor^TM^ did not give reasonable enantioselectivities [81], the pyridinium salt **6** turned out to be very promising, thus suggesting that ion pairing between the chiral phosphate **G1** and **6** should play a fundamental role herein. In 2014, Akiyama’s group then reported the use of chiral sodium phosphates for this α-fluorination reaction [82]. Here it was clearly shown that the phosphoric acid **H** alone does not sufficiently catalyse and control the reaction. However, addition of Na_2_CO_3_ (leading to formation of the sodium enolate of **1** and the sodium phosphate of **H**) had a very pronounced effect on both, yield and enantioselectivity when using NFSI as the fluorinating agent. Again, the exact mechanistic understanding of this reaction is still rather difficult and also subject to speculation, but Akiyama and co-workers provide a very plausible scenario for the high selectivity of their catalyst system in their original contribution [82].

**Scheme 5:**
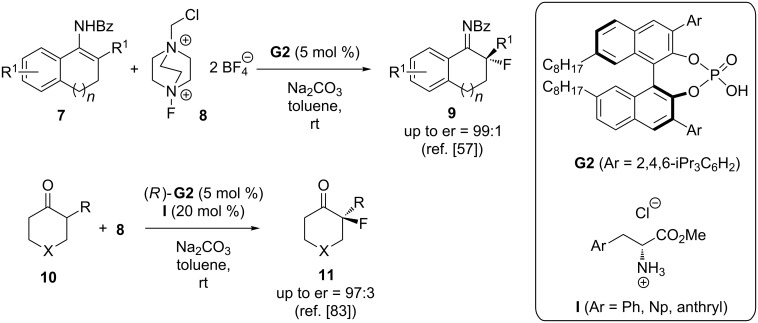
Anionic PTC-catalysed α-fluorination of enamines **7** and ketones **10**.

Toste’s group has contributed to the development of asymmetric anionic phase-transfer catalysis like no others over the last years (for some interesting examples please see [56–59,61,63]). Very impressively, they succeeded in introducing a rather generally applicable system to control the stereochemical outcome of a variety of challenging reactions that use Selectfluor^TM^ (**8**) as a simple and highly reactive electrophilic F-transfer reagent. Besides fluoro-cyclizations of olefines [56], also the asymmetric α-fluorination of prochiral carbonyl compounds or analogues like enamides **7** has been carried out with remarkable enantioselectivities by using phosphoric acid **G2** under basic conditions (leading to the sodium salt of **G2** which then acts as the PTC in this reaction) [57] (Scheme 5). The authors propose a bifunctional activation mode of the catalyst for this reaction by simultaneously coordinating the cation **8** via ion pair formation and by H-bonding between the phosphate moiety and the enamide [57], which gives a very convincing explanation for the observed stereochemical outcome of this reaction. In 2014, the same group then succeeded in carrying out this transformation by starting directly from ketones **10** [83]. Crucial to access high yields and high selectivities was the use of a dual catalyst system consisting of **G2** as the anionic PTC and amino acids **I** for in situ formation of a more reactive enamine intermediate which then undergoes the stereoselective α-fluorination followed by hydrolysis of the resulting imines to give targets **11** [83].

#### α-Chlorinations

The stereoselective synthesis of chiral α-chlorinated carbonyl compounds is an important topic, since these targets can be valuable intermediates for the synthesis of a variety of different biologically active molecules [85–86]. The main interest in these compounds comes from their versatility for further functionalizations by stereospecific nucleophilic S_N_2-type displacement reactions with different nucleophiles [66,85–87]. Accordingly, it comes as no surprise that their asymmetric synthesis has been intensively investigated in the past, either relying on asymmetric (transition)-metal- or organo-catalysis [76,85–94]. In sharp contrast to the numerous reports describing the use of asymmetric phase-transfer catalysis for α-fluorination reactions (as stated above), the use of chiral PTCs for enantioselective α-chlorinations of prochiral nucleophiles has been much less systematically investigated so far [76,90,92].

**Scheme 6:**
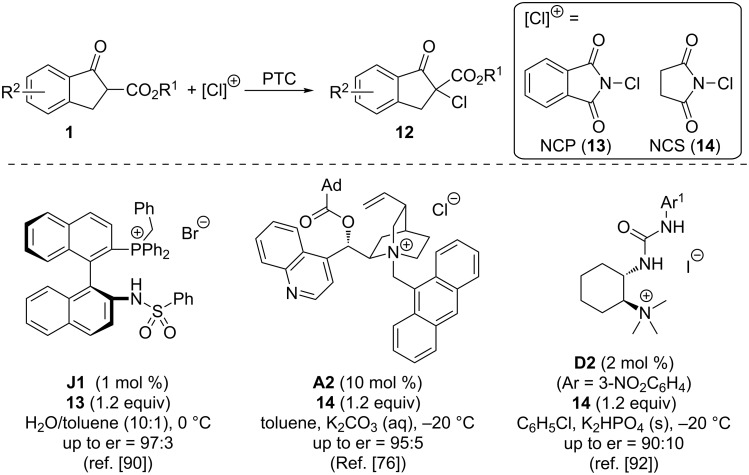
PTC-catalysed α-chlorination reactions of β-ketoesters **1**.

In 2013, Maruoka and co-workers introduced a new family of bifunctional phosphonium salts **J** which turned out to be highly active catalysts for different α-heterofunctionalization reactions of ketoesters **1** under base-free conditions [90]. Key to success in this spectacular report was the use of the systematically optimized sulfonamide-containing phosphonium salt **J1**. With this catalyst, they were able to achieve high selectivities in the α-chlorination of **1** with *N*-chlorophthalimide (**13**) as the Cl-source under H_2_O-rich conditions, as outlined in Scheme 6. Around the same time, the groups of Meng and Lu also demonstrated that the cinchona alkaloid-based catalyst **A2** not only allows them to carry out the above described asymmetric α-fluorination reactions (Scheme 2), but also holds promise for the highly enantioselective α-chlorination of β-ketoesters **1**, by using *N*-chlorosuccinimide (**14**) as the Cl-transfer agent [76]. In continuation of our efforts to develop bifunctional ammonium salts **D** for asymmetric catalysis we have also recently investigated the asymmetric synthesis of α-chloroketoesters **12** [92]. We found that catalyst derivative **D2** performs best for this purpose, even with rather low catalyst loadings, but it has to be admitted that in general the enantioselectivities are lower than in the two other case studies shown in Scheme 6 [92].

#### α-Bromination and α-iodination reactions

Asymmetric α-bromination and α-iodination reactions of prochiral nucleophiles have been much less commonly reported than fluorination and chlorination reactions [49–54] and only a few reasonably enantioselective organocatalytic approaches have been reported so far [54,95–96]. In these few reports it became obvious that it is a much bigger challenge to achieve an efficient stereodifferentiation in the C–Br or C–I bond formation compared to the analogous α-chlorinations and α-fluorinations. To the best of our knowledge no asymmetric phase-transfer catalysed approach for such reactions has been reported so far. We have recently investigated the phase-transfer catalysed α-cyanation of ketoesters **1** using hypervalent iodine-based cyanide transfer reagents [97]. Hereby we observed the in situ oxidation of the PTC counter anions (Br^−^ or I^−^) and subsequent α-halogenation of **1**. We tried to employ and optimise this procedure, but without any success in terms of enantioselectivity as under no conditions any asymmetric induction could be observed and the same was the case when we tested the use of *N*-bromosuccinimide under PTC conditions with a variety of different chiral catalysts, thus illustrating rather well some of the major present limitations of asymmetric phase-transfer catalysis. Hence, it is without doubt that the introduction and development of such highly selective transformations will be one of the important and challenging targets in the future.

### α-Hydroxylations and oxygenations

The stereoselective formation of a C–O bond in the α-position of a carbonyl (or analogues) group is an important transformation, mainly because the hereby accessed α-hydroxylated/oxygenated targets are frequently found as such, or in a masked form, in natural products or biologically active pharmaceutical or agrochemical lead structures [98–101]. Several strategies to access these valuable motives in an enantioenriched fashion have been reported in the past. One option would be to use already α-functionalized (i.e., halogenated) carbonyl compounds and carry out functional group interconversions, like, e.g., stereospecific nucleophilic displacement reactions [87]. Another powerful strategy would be the use of prochiral α-halocarbonyl compounds for asymmetric Darzens-type reactions to obtain chiral α,β-epoxycarbonyl compounds, which has been rather successfully demonstrated under chiral phase-transfer catalysis in the past [18,102–104].

Besides those methods that make use of already α-functionalized carbonyl compounds, the direct stereoselective α-oxygenation or α-hydroxylation of simple prochiral nucleophiles with either oxygen as such, or an electrophilic oxygen species became by far the most important and most thoroughly investigated strategy. Hereby both, approaches relying on either asymmetric metal- or organocatalysis, have been well-investigated already [105–122]. In the field of non-covalent asymmetric organocatalysis, chiral H-bonding catalysis [37,121–122] and chiral phase-transfer ion-pairing catalysis [110–119] turned out to be extremely powerful.

**Scheme 7:**
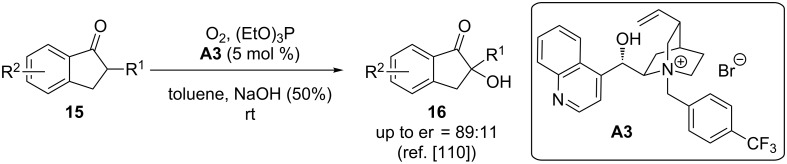
Shioiri’s seminal report of the asymmetric α-hydroxylation of **15** with chiral ammonium salt PTCs.

Very remarkably, in 1988 Shioiri’s group already described the asymmetric synthesis of differently substituted α-hydroxy ketones **16** under chiral phase-transfer catalysis [110]. In this seminal investigation, they succeeded in carrying out the direct α-hydroxylation of simple ketones **15** by using O_2_ in combination with triethylphosphite, which leads to the in situ formation of a reactive hydroperoxide derivative. When using the easily accessible cinchona alkaloid-based PTC **A3**, the chiral products **16** were obtained in high enantioselectivities under operationally simple biphasic reaction conditions, as outlined in Scheme 7.

**Scheme 8:**
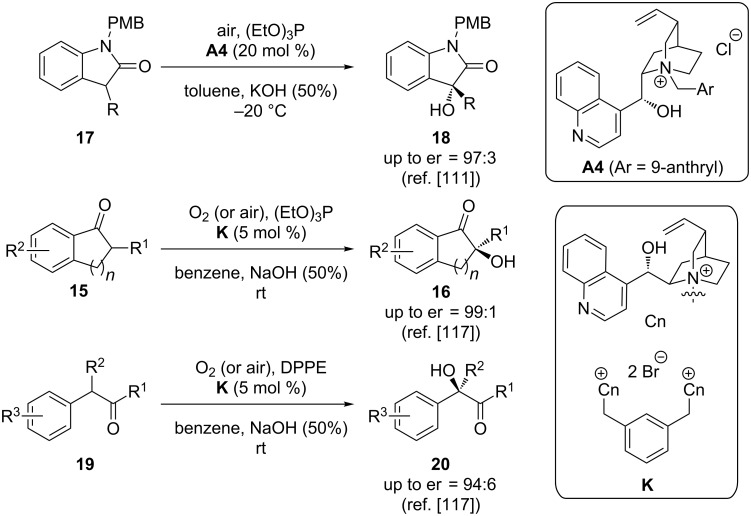
Asymmetric ammonium salt-catalysed α-hydroxylation using oxygen together with a P(III)-based reductant.

Rather surprisingly, despite this pioneering early report almost 20 years passed until this methodology became more systematically investigated. In 2008, Itoh’s group described the α-hydroxylation of oxindoles **17** under phase-transfer conditions by using air in combination with (EtO)_3_P as the oxygenation system (Scheme 8, upper reaction) [111]. Very recently, Zhao et al. then developed this elegant methodology further by identifying the dimeric cinchonine (Cn)-based ammonium salt **K** as the PTC of choice for the highly enantioselective direct α-hydroxylation of cyclic and acyclic ketones **15** and **19** by using oxygen or air together with either (EtO)_3_P or DPPE (1,2-bis(diphenylphosphino)ethane) as a phosphorous(III)-based reductant for the oxygen-transfer (Scheme 8, lower part) [117].

**Scheme 9:**
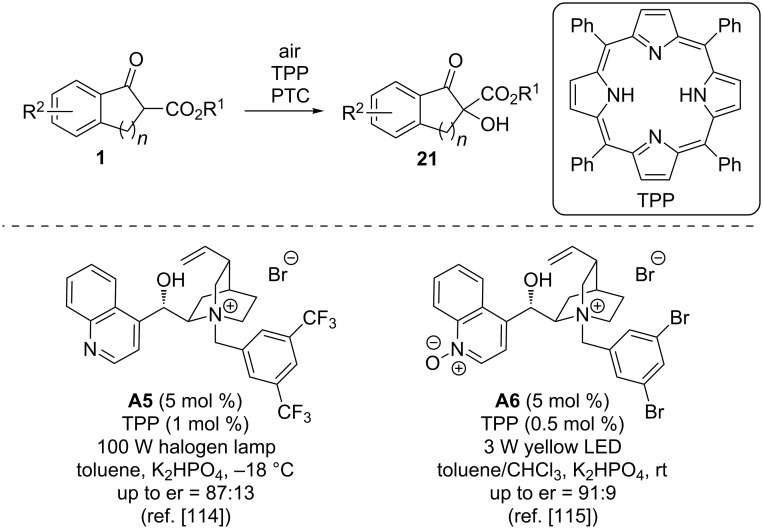
Asymmetric ammonium salt-catalysed α-photooxygenations.

Besides making use of oxygen or air together with a stoichiometric activator/reductant as described above (Scheme 7 and Scheme 8), the photooxygenation of prochiral substrates like β-ketoesters **1** with O_2_ or air in the presence of a chiral PTC and TPP (tetraphenylporphyrin) as a photosensitizer has recently been reported to proceed with satisfying selectivities by the groups of Meng and Gao [114–115]. In their first report [114], they made use of the classical cinchona catalyst **A5** together with catalytic amounts of TPP under irradiation with a 100 W halogen lamp with air as the oxygen source. Very recently, they then introduced the *N*-oxide-containing PTC **A6**, which gave even higher selectivities and was successfully used under yellow LED (3 W) irradiation [115] (Scheme 9).

**Scheme 10:**
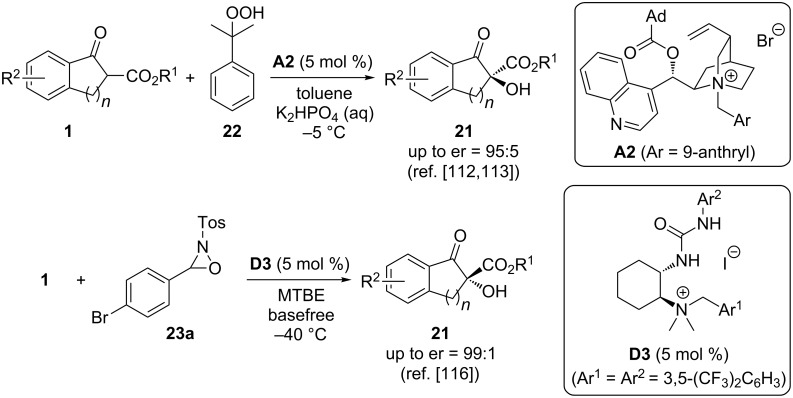
Asymmetric ammonium salt-catalysed α-hydroxylations using organic oxygen-transfer reagents.

All the approaches described so far made use of O_2_ or air as the oxygen source, which is of course the most economical way of carrying out oxidations. However, there have also been several rather successful and highly enantioselective reports that describe analogous α-hydroxylation reactions by using alternative oxygen-transfer reagents (Scheme 10). A few years ago, Meng et al. carried out the α-hydroxylation of substrates **1** by using cumyl hydroperoxide (**22**) as an easily available oxidizing agent, which worked well to access a series of differently substituted products **21** in high yields and with high enantioselectivities when using the carefully optimized sterically demanding PTC **A2** [112–113]. Our group has very recently investigated the use of bifunctional catalysts **D** for such α-hydroxylations [116]. Hereby we realized that oxaziridines like compound **23a** are versatile reagents for this reaction, giving access to products **21** with excellent enantioselectivities under base-free conditions (for selected other uses of oxaziridines in asymmetric α-hydroxylation reactions please see [123–124]). Interestingly, this transformation is accompanied by a kinetic resolution of the employed oxaziridine with *s*-factors up to 45 [116].

**Scheme 11:**
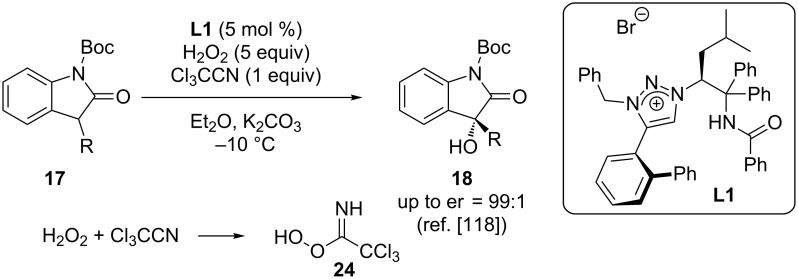
Asymmetric triazolium salt-catalysed α-hydroxylation with in situ generated peroxy imidic acid **24**.

One potentially useful simple reagent to carry out oxygen-transfer reactions is hydrogen peroxide (H_2_O_2_). Unfortunately, the direct use of this base-chemical under asymmetric organocatalysis turned out to be rather tricky for α-hydroxylation reactions. One recent report by the Ooi group overcame some of the limitations by using H_2_O_2_ in combination with trichloroacetonitrile (Cl_3_CCN) [118]. This combination leads to the in situ formation of peroxy imidic acid **24**, which then serves as the O-transfer reagent for the asymmetric α-hydroxylation of oxindoles **17** in the presence of the chiral triazolium-based ion pairing catalyst **L1** (Scheme 11).

Very recently, Toullec and co-worker reported the use of phase-transfer catalysts to carry out the dearomatization of phenol and naphthol derivatives **25** via *ortho*-hydroxylation to obtain the highly-functionalized targets **26** [119]. Hereby oxaziridines **23** were found to be the best-suited hydroxylating agents. Unfortunately, the reaction has so far only been successful in a racemic fashion, whereas the use of chiral PTCs like **A7** gave very little enantioselectivity only (er = 56:44, Scheme 12).

**Scheme 12:**
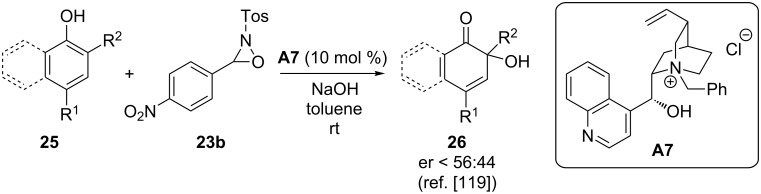
Phase-transfer-catalysed dearomatization of phenols and naphthols.

Nevertheless, this inspiring report very nicely demonstrated the potential of phase-transfer catalysis for such dearomatization reactions and it is without doubt that it will inspire other groups to enter the field as well.

**Scheme 13:**
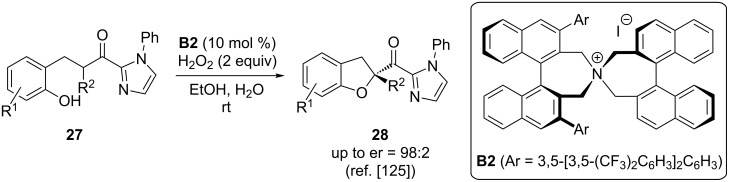
Ishihara’s ammonium salt-catalysed oxidative cycloetherification.

A very remarkable strategy for the α-oxygenation of prochiral nucleophiles was recently reported by Ishihara and co-workers [125–126]. They succeeded in developing a highly enantioselective protocol for oxidative cycloetherification reactions based on the use of in situ-generated chiral quaternary ammonium hypoiodite species. By using Maruoka-type catalysts **B**, which contain an iodide counter anion, in the presence of a simple oxidant like H_2_O_2_ they were able to generate a chiral hypoiodite species in situ, which then facilitates the asymmetric intramolecular cycloetherification of starting materials **27** (Scheme 13). One of the important observations in this impressive report was the necessity of the imidazole moiety to achieve high selectivities [125]. In a subsequent study, the same group also expanded this concept to 6-ring forming cycloetherifications, which gave a straightforward access to tocopherols with very high selectivities [126].

### α-Thioetherifications

**Scheme 14:**
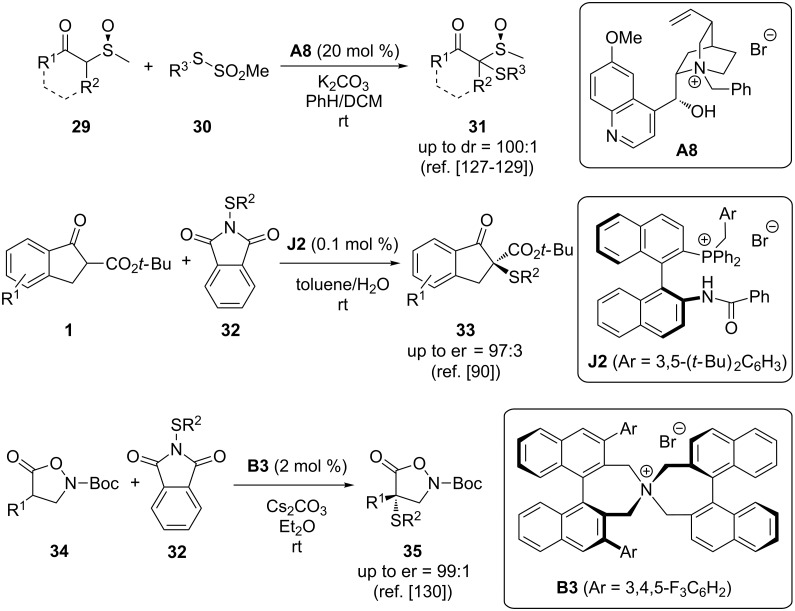
Chiral phase-transfer-catalysed α-sulfanylation reactions.

The catalytic stereoselective C–S bond formation in the α-position of prochiral nucleophiles became the topic of broader interest rather recently [127–134]. Again, different catalytic strategies have been successfully employed to achieve these transformations and asymmetric phase-transfer catalysis is one powerful option to control the configuration of the newly installed C–S bond. The first reports describing the use of phase-transfer catalysis for α-sulfenylation reactions date back almost 20 years, when Wladislaw and co-workers described the diastereoselective α-sulfenylation of chiral β-ketosulfoxides **29** in the presence of achiral and chiral PTCs (Scheme 14, upper reaction) [127–129]. In a detailed investigation of different cyclic and acyclic sulfoxides **29**, they could show that the diastereoselectivity of this reaction can be clearly improved by using the chiral cinchona alkaloid-based ammonium salt **A8** compared to the use of achiral tetraalkylammonium salt-based PTCs [127–129]. In 2013, Maruoka’s group then described the use of the bifunctional phosphonium salt-based catalyst **J2** for the α-sulfanylation of β-ketoesters **1** [90]. Very impressively, only 0.1 mol % of the catalyst were sufficient to achieve high yields and high enantioselectivities for this transformation under base-free water-rich conditions (Scheme 14, second reaction). Very recently, Briere and co-workers investigated the asymmetric α-sulfanylation of isoxazolidinones **34** [130]. This approach gave access to a variety of chiral α-sulfanyl-β^2,2^-amino acid precursors **35** in a straightforward and so far unprecedented fashion. The reaction was tested in the presence of cinchona alkaloid based catalysts **A** and Maruoka-type catalysts **B**. The latter were found to be significantly more promising and, after some fine-tuning, the use of catalyst **B3** allowed them to access the targets **35** with very high enantioselectivities (Scheme 14, lower reaction) [130].

**Scheme 15:**
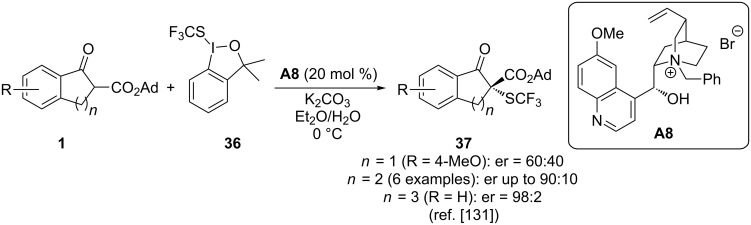
Chiral phase-transfer-catalysed α-trifluoromethylthiolation of β-ketoesters **1**.

Besides these alkyl- or aryl-thioether-forming reactions depicted in Scheme 14, also the introduction of a CF_3_S group to access trifluoromethylthioethers has been recently investigated [131]. In a very detailed study, Shen and co-workers have investigated the use of different cinchona alkaloid-based organocatalysts to carry out the α-trifluoromethylthiolation of β-ketoesters **1** by using the hypervalent iodine-based CF_3_S-transfer reagent **36** in an asymmetric fashion. Very interestingly, they realized that for indanone-based ketoesters **1** (with *n* = 1) simple cinchona alkaloids themselves gave the products in high yields and with high selectivities, whereas PTCs performed less selective herein. However, for tetralone-based starting materials **1** (with *n* = 2) or even larger ring-containing esters **1** (*n* = 3) these simple alkaloids did not allow them to access the products in more than 10% yields. This obstacle could, however, be overcome by using chiral PTCs **A** instead, which in that cases allowed them to access the larger ring-sized products **37** in satisfying yields and with high enantioselectivities [131] (Scheme 15).

### α-Aminations

The α-C–N bond formation of prochiral nucleophiles is one of the essential transformations in (bio)organic chemistry and the importance of the hereby accessed chiral α-aminocarbonyl compounds like, e.g., α-amino acids or others cannot be overestimated [135]. Accordingly, it comes as no surprise that the development of asymmetric catalytic methods towards these important targets has been heavily investigated in the past [136–146] and asymmetric phase-transfer catalysis turned out to be highly promising herein too [138–145]. Similar as described for phase-transfer-catalysed α-oxygenation reactions above, also the introduction of a nitrogen-based functionality alpha to a carbonyl group can be achieved by several complementary strategies. One powerful option is to carry out Michael addition-initiated aziridination reactions of α,β-unsaturated carbonyl acceptors [18,138–140]. Another possibility to access aziridines would be to carry out aza-Darzens type reactions, which, however, is yet a rather difficult transformation under asymmetric phase-transfer catalysis [18]. Again, one of the most straightforward approaches is to start from relatively simple prochiral carbonyl precursors and carry out the direct α-amination with a suitable electrophilic N-transfer reagent in the presence of a chiral catalyst to ensure an efficient face-differentiation in the C–N bond formation. This strategy has been investigated under chiral phase-transfer catalysis in the past and the results have been rather promising, as will be discussed in the following chapter [61,138–140].

**Scheme 16:**
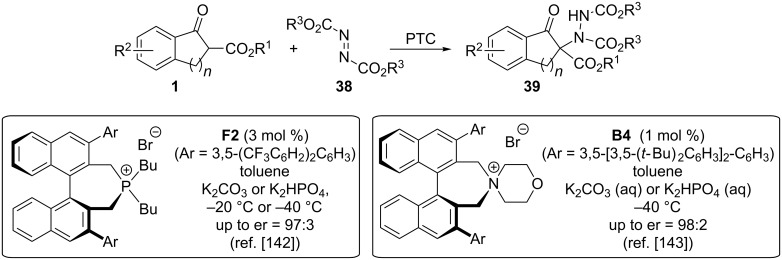
Chiral phase-transfer-catalysed α-amination of β-ketoesters **1** using diazocarboxylates **38**.

One of the key-questions in this field is the nature of the electrophilic N-transfer reagent. One class of reagents that has been employed for α-amination reactions for almost one century now are azodicarboxylates **38** [147]. Addition of a nucleophile like β-ketoesters **1** to **38** gives the corresponding hydrazides **39**, which can then be further manipulated with established methods [137,142–143]. In 2008, Maruoka’s group reported the use of the chiral phosphonium salt **F2** as a phase-transfer catalyst for the α-amination of **1** with different diazocarboxylates **38** (Scheme 16) [142,144]. This report is not only remarkable for the high selectivities and the fact that the hereby accessed products **39** can then serve as valuable intermediates for further transformations like, e.g., the synthesis of the aldose reductase inhibitor Ranirestat, but also because it presents an early and pioneering report on the use of chiral tetraalkylphosphonium salts as asymmetric PTCs. Just a year later, the same group then also succeeded in using the chiral spirocyclic quaternary ammonium salts **B** for the same transformation, which performed even slightly more selective with low catalyst loadings of 1 mol % only [143].

**Scheme 17:**
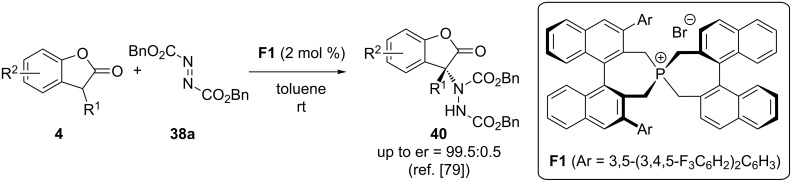
Asymmetric α-fluorination of benzofuranones **4** using diazocarboxylates **38** in the presence of phosphonium salt PTC **F1**.

As discussed before already, the spirocyclic phosphonium salt **F1** was recently used as a phase-transfer catalyst for asymmetric α-fluorination reactions by the groups of Cahard and Ma (Scheme 3) [80]. The same groups also investigated the use of this catalyst class for asymmetric α-amination reactions of benzofuranones **4** with diazocarboxylates **38** [79]. Very interestingly, the reaction could be carried out under base-free conditions to give the products **40** with excellent enantioselectivities (Scheme 17). Detailed mechanistic studies suggest that catalyst **F1** favours enol formation of starting material **4**, thus providing a convincing rational why no base is required for this transformation [79].

**Scheme 18:**
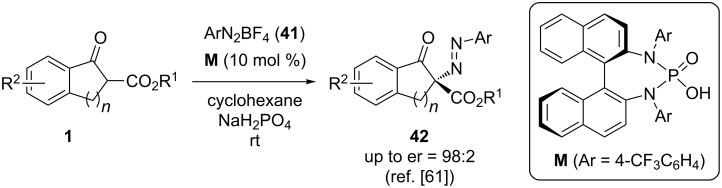
Anionic phase-transfer-catalysed α-amination of β-ketoesters **1** with aryldiazonium salts **41**.

Another class of easily accessible electrophilic N-transfer reagents are (aryl)-diazonium salts like compounds **41**. The control of these cationic reagents under chiral phase-transfer catalysis was recently accomplished by Toste’s group using chiral anion-based PTCs [61]. In this seminal report, they realized that commonly used phosphoric acids like compounds **G** (see Scheme 5) do not result in satisfying selectivities. Impressively, however, changing for BINAM-based phosphoric acid amides like compound **M** they achieved remarkable enantioselectivities for this transformation (Scheme 18). It was also demonstrated that the hereby accessed products **42** can easily be reduced to obtain the corresponding free amines by means of a palladium-catalysed heterogeneous hydrogenation [61].

**Scheme 19:**
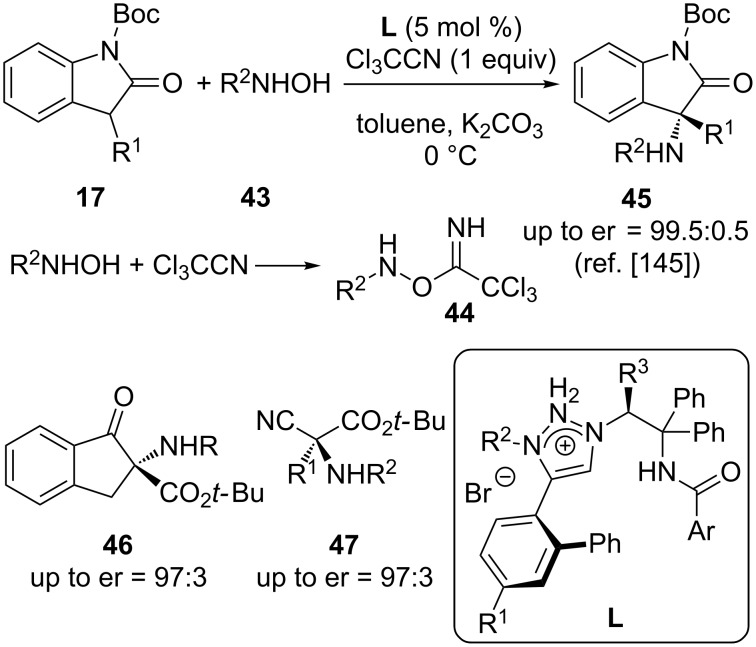
Triazolium salt **L**-catalysed α-amination of different prochiral nucleophiles with in situ activated hydroxylamines **43**.

In continuation of their elegant work on generating electrophilic heteroatom-transfer reagents in situ upon adding a heteroatom nucleophile to trichloroacetonitrile (compare with Scheme 11), Ooi’s group has recently also expanded this powerful concept to asymmetric α-amination reactions [145]. By using hydroxylamines **43** as simple N-containing reagents, the addition of these compounds to trichloroacetonitrile gives the reactive intermediates **44**, which then serve as versatile electrophilic N-transfer reagents under asymmetric triazolium salt **L** catalysis (Scheme 19). A variety of different prochiral nucleophiles were successfully employed and thanks to the modular synthesis of catalysts **L** a straightforward and rapid catalyst fine-tuning for every given target reaction could be achieved, thus making this concept highly promising for future applications.

Despite of all these recent achievements, it is yet fair to say that compared to other α-heterofunctionalization reactions the asymmetric PT-catalysed α-amination has so far been surprisingly less systematically explored and in the authors view, this transformation is still far from being a solved problem, especially when it comes to the synthesis of unprotected α-aminocarbonyl targets.

**Scheme 20:**
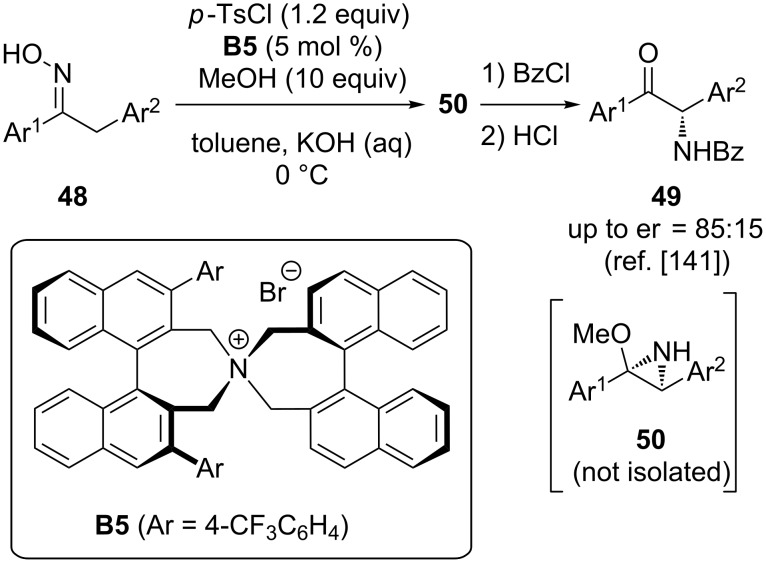
Phase-transfer-catalysed Neber rearrangement.

One conceptually alternative and long-known approach to access chiral α-aminocarbonyl compounds is to start from simple oximes **48** and carry out a Neber rearrangement [148]. Very remarkably, in 2002 already, Maruoka and co-workers demonstrated the potential of asymmetric cation-based phase-transfer catalysis to control the absolute configuration of the newly-installed stereogenic centre in this rearrangement reaction [141]. By using their spiro-quaternary ammonium salt catalyst **B5** they were able to control the Neber rearrangement oximes **48** to the α-amino ketones **49** (proceeding via the not-isolated intermediate **50**) with reasonable enantioselectivities up to 85:15 (Scheme 20). Although the selectivities were not perfectly satisfying and the application scope limited to two examples, this early reported clearly demonstrated the high potential of asymmetric PTCs to control reactions that proceed via anionic intermediates and that are only hardly possible with other catalytic methods.

## Conclusion

Chiral phase-transfer catalysis has emerged as a very powerful method in asymmetric catalysis over the last decades and numerous highly enantioselective approaches have been reported so far, which clearly proves the potential of this non-covalent activation principle. Besides versatile stereoselective C–C bond-forming reactions, the asymmetric α-heterofunctionalization of prochiral nucleophiles (i.e., enolates) became one of the privileged application fields for this catalysis concept. We hope that we could provide an illustrative overview about the major recent achievements in this field (either relying on the use of chiral cation- or anion-based PTCs). Despite these impressive examples, it is, however, also fair to say that in our opinion this field still requires further improvements. By looking for example on the difficulties in achieving high selectivities for asymmetric α-bromination or α-iodination reactions, it becomes clear that new methods are urgently required. Another application that may become of future interest could be asymmetric α-phopshorylation approaches or the introduction of other heteroatoms (e.g., boron), which are all transformations that are without doubt important, but so far much less systematically addressed as most of the examples we showed herein. We are therefore convinced that the field of asymmetric phase-transfer catalysis will be a heavily investigated one further on, and are confident that it will significantly contribute to the advancement of chiral α-heterofunctionalization reactions in the future.
